# Relationship between School Integration, Psychosocial Adjustment and Cyber-Aggression among Adolescents

**DOI:** 10.3390/ijerph18010108

**Published:** 2020-12-26

**Authors:** Celeste León Moreno, Gonzalo Musitu Ochoa, Elizabeth Cañas Pardo, Estefanía Estévez López, Juan Evaristo Callejas Jerónimo

**Affiliations:** 1 Department of Education and Social Psychology, Pablo Olavide University, 41013 Seville, Spain; gmusoch@upo.es (G.M.O.); jecaljer@upo.es (J.E.C.J.); 2 Department of Health Psychology, Miguel Hernández University, Avda. de la Universidad s/n, 03202 Alicante, Spain; elizabeth.canas@goumh.umh.es (E.C.P.); eestevez@umh.es (E.E.L.)

**Keywords:** school integration, psychosocial adjustment, cyber-aggression, adolescence

## Abstract

The aim of this study was to analyze the relationships between sociometric types in the classroom—rejected, preferred, neglected, controversial and average—and psychological discomfort, life satisfaction and cyber-aggression, based on the adolescent’s gender. 2398 adolescents of both sexes participated in the study (49.8% girls), aged between 12 and 18 years (*M* = 16.03, *SD* = 1.91). Multivariate analyses of variance were performed. The results showed significant relationships between sociometric types, life satisfaction and cyber-aggression. Rejected adolescents also showed less satisfaction with life and greater cyber-aggression. Furthermore, the boys, regardless of their sociometric type in the classroom, displayed less psychological distress and less involvement in cyber-aggression. Controversial adolescents also showed greater involvement in cyber-aggression. Finally, programs should be promoted for the prevention of social difficulties in the school, based on the promotion of social integration, not only in the classroom, but also on the Internet.

## 1. Introduction

### 1.1. School Integration

Problems of school integration among adolescents have received special attention from researchers, teachers and school leaders due to their serious consequences for the emotional well-being and psychosocial adjustment of students [1,2]. In this sense, numerous studies have associated the social integration difficulties manifested by some students with lower self-esteem [3,4], greater likelihood of depression and anxiety [5,6], higher rates of absenteeism and school dropout [7], serious problems of violence and aggressive behaviors in the classroom [8,9] and even suicidal ideation [10].

One of the most used criteria for determining student integration in the classroom are sociometric measurements in which the acceptance and rejection relationships between classmates are evaluated based on a pre-established criterion (e.g., sympathy or group work) [11]. This technique analyzes group organization and the position occupied in same by each student based on the choices and rejections made with respect to their peers [12]. Five sociometric types are obtained from the combination of these scores: rejected, preferred, neglected, controversial and average [11,13].

Rejected: these are students who are disliked by most of their peers. These adolescents are more frequently involved in violent and disruptive behaviors, report more conflictive relationships with peers and with teachers, and show low social and academic competence [8]. However, various studies have shown that not all rejected adolescents participate in violent behavior and that violence is therefore not necessarily associated with rejection [14,15]. A proportion of rejected students display excessive social withdrawal, depression and anxiety, an aspect that can also contribute to the adolescent being rejected, and to remaining in this sociometric type [9].

Preferred: these are students who are liked by most of the group. Compared to other adolescents, preferred students tend to show greater social competence and cognitive skills (e.g., companionship, sympathy, good character) [3] and lower levels of aggressive and transgressive behaviors [16]. Students preferred by their peers tend to have greater social skills for achieving interpersonal goals and at the same time maintain positive social relationships, compared to the other sociometric types [17].

Neglected: these are students who are subject to indifference from their peer group, receive little attention from their peers and are not well known. These adolescents are quiet, shy and reserved, but they do not necessarily have to be socially isolated like some rejected adolescents [18]. Although they display less sociability than average peers, they respect the rules and are engaged in socially accepted activities, albeit to a lesser degree than more accepted children and in a more isolated manner [19].

Controversial: these students are characterized by having high levels of acceptance and rejection, i.e., they are liked by many, but also disliked by many. This sociometric type also presents behaviors common to both groups (preferred and rejected), since they are highly involved in both prosocial and antisocial behaviors [18].

Average: these are students who, compared to their peer group, do not stand out for being especially accepted or rejected by their peers. These adolescents do not seem to stand out, although they are more visible than neglected students, so they do not report very high scores with respect to either the positive traits characteristic of preferred adolescents or the negative traits that characterize rejected teens [18].

### 1.2. Psychosocial Adjustment and School Integration

The degree of social acceptance and rejection experienced by adolescents are key aspects for psychosocial adjustment at this stage of life. Students with classroom integration problems often report low levels of emotional well-being [5,20]. Numerous findings suggest that the lack of social acceptance by peers is a traumatic experience, associated with a negative assessment of one’s life [21,22] and with greater emotional distress [4,23]. The lack of social acceptance by peers has also been linked to externalizing problems in adolescence [24,25], higher levels of anxiety [26], anger and hostility [6]. Some authors also report that adolescents who are less integrated in the classroom are precisely the ones more frequently involved in violent behavior towards their peers in the school environment [27,28]. However, and despite empirical evidence showing that online adolescents perpetuate the social relations that already exist in the classroom [29], the number of studies specifically linking sociometric types with cyber-aggression is still small.

### 1.3. Cyber-Aggression and School Integration

Cyber-aggression is defined as an aggressive, intentional act carried out by a group or an individual, using electronic forms of contact, repeatedly and over time against a victim who cannot easily defend him or herself [10,30]. In many cases, there is a continuity between aggression at school and cyber-aggression. Thus, an adolescent’s problems in school are transferred to and continue on the Internet [31]. Previous studies have reported that rejected students use aggressive strategies when interacting with their peers online [32,33], transforming them in turn into adolescents with low social support [28].

In terms of gender-based differences, it has been observed that girls tend to establish friendships with fewer peers [34]. They establish more intense and intimate friendships, while boys prefer to establish relationships with a larger number of peers [35]. Boys also state that their friendships are characterized by less expression of personal feelings and greater difficulties in conflict resolution [36,37]. Likewise, it has been observed that rejected and neglected students are mostly male, while average and preferred students are mainly female [7]. It has also been observed that girls are more likely to experience emotional well-being problems [38,39], although some studies have reported a similar trend in both boys and girls [40,41,42]. One controversial aspect concerns the prevalence of cyber-aggression according to gender. Some authors have observed that cyber-aggression is more common in boys [43], while others have found that it is a more frequent behavior in girls [44]. Other authors have even concluded that involvement in cyber-aggression is similar in boys and girls [45,46].

Despite the relevance of the previously described variables, very few studies have analyzed the role played by different sociometric types of student in the classroom in the relationship between psychosocial adjustment, life satisfaction and cyber-aggression. Most research in this field focuses on the psychological and social consequences of school rejection [3,4,6]. This study aims to analyze the relationships between sociometric types in the classroom, rejected, preferred, neglected, controversial and average, with psychosocial adjustment, life satisfaction and cyber-aggression based on the adolescent’s gender. Based on this objective, the following hypotheses were considered: (H1) Students with school integration problems (rejected and neglected) will show greater psychological distress and less satisfaction with life, (H2) Students with school integration problems (rejected and neglected) will obtain higher scores in cyber-aggression; (H3) The rejected and neglected sociometric types will be associated with greater psychological distress and involvement in cyber-aggression, and less satisfaction with life in boys, compared to girls.

## 2. Materials and Methods

### 2.1. Participants

The sample comprised 2398 adolescents of both sexes (49.8% girls) aged between 12 and 18 years (M = 16.03, SD = 1.91) who were studying Compulsory Secondary Education (ESO) in nine centers in Andalusia (Spain). The participants attended public (56.4%) and semi-private schools (43.5%). The participants were selected by means of stratified random sampling. The sampling units were geographic area (urban or rural) and school ownership (public or private). The size required for this sample with a sampling error of ±2%, a confidence level of 95% and a population variance of 0.50, was 2398 adolescents. Lost data by scales or sub-scales, provided they did not exceed 15%, were processed using the multiple linear regression imputation model [47]. Univariate atypical data were detected by the exploration of standardized scores [48].

### 2.2. Procedure

After obtaining permission from the schools and the active consent of the families to carry out the research, the battery of instruments was administered under the supervision of the research staff in two different sessions of approximately 45 min in school hours. To complete the sociometric questionnaire, the students were given a numbered class list to write down the numbers assigned to their classmates. Each student was given a list and instructed not to disclose their responses. The study complied with the ethical principles established by the Helsinki Declaration (1964): protection of personal data and guarantees of confidentiality, informed consent and the right to information, free consent, non-discrimination, and the possibility of abandoning the study in any of the phases.

### 2.3. Instruments

Sociometric type. The sociometric questionnaire was used [49]. This questionnaire consists of 4 items that, based on the “classmate” criterion, allowed the following aspects to be measured: (a) positive choices (“Who would you choose as classmates?”); (b) negative choices (“Who would you NOT choose as classmates?”); (c) positive perceptions (“Who do you think would choose you as a classmate?”); and (d) negative perceptions (“ Who do you think would NOT choose you as a classmate?”). The choices were limited to three students and the order of preference was weighted.

Psychological distress. The Psychological Distress Scale was used [50]. The scale consists of 10 Likert-type items with five response options (1 = never, 5 = always) that assess depressive and anxiety symptoms (e.g., “How often did you feel depressed”), Ω = 0.91, α = 0.87. The confirmatory factor analysis (CFA) showed a good fit of the model to the SB (Satorra-Bentler) dataχ^2^ = 309.7391, df = 30, *p* < 0.001, CFI = 0.960, RMSEA = 0.062, I.C. 90 (0.056, 0.069).

Satisfaction with life. The Satisfaction with Life Scale [51], adapted to Spanish by Atienza et al. (2000), was used [52]. This scale consists of 5 items with four response options (1 = strongly disagree and 5 = strongly agree) that provide a general index of satisfaction with life relating to the subjective well-being perceived by the adolescent (e.g., “In most ways, my life is close to my ideal”), Ω = 0.83, α = 0.76. The confirmatory factor analysis (CFA) showed a good fit of the model to the SB (Satorra-Bentler) dataχ^2^ = 30.4954, df = 5, *p* < 0.001, CFI = 0.988, RMSEA = 0.046, I.C. 90 (0.031, 0.062).

Cyber-aggression. The Adolescent Aggression through Mobile Phone and Internet Scale (CYB-AG: [53]) was used. This scale consists of 10 items with five response options (1 = never and 5 = often) that assess the frequency of participation in aggressive behaviors through new technologies in the last 12 months (e.g., “I have sent or tampered with someone’s photos or videos without their permission”), Ω = 0.83, α = 0.81. The confirmatory factor analysis (CFA) showed a good fit of the model to the SB (Satorra-Bentler) dataχ^2^ = 53.8750, df = 33, *p* < 0.05, CFI = 0.978, RMSEA = 0.016, I.C. 90 (0.008, 0.024).

### 2.4. Data Analysis

First, the basic structure of relationships in the classroom group was analyzed with the SOCIOMET program [54]. This software analyzes group organization and the position occupied in same by each student based on the choices and rejections made with respect to their peers. The five sociometric types were obtained from the combination of these scores [3]: (1) rejected (they receive a significantly high number of rejections), *n* = 273; (2) preferred (they receive a significantly high number of choices from their peers), *n* = 188; (3) neglected (the number of choices and rejections are significantly low), *n* = 291; (4) controversial (they receive a high number of elections, but also rejections), *n* = 86; (5) average (cases not assigned to any of the previous categories), *n* = 1560 (see Table 1).

Subsequently, a multivariate factorial design (MANOVA, 5 × 2) was carried out with the SPSS statistical analysis program (version 20) considering the sociometric types (rejected, preferred, neglected, controversial and average) and gender (boys versus girls) as fixed factors to analyze potential interaction effects. Psychological distress, life satisfaction and cyber-aggression were treated as dependent variables. Then, univariate tests (ANOVAS) were performed to analyze the statistically-significant differences in the variables and the Bonferroni post-hoc test was applied (α = 0.05).

## 3. Results

In the MANOVA, statistically significant differences were found in the main sociometric effects (Λ = 0.985, *F*(12, 6313) = 1801, *p* < 0.001, η^2^p = 0.005) and gender (Λ = 0.981, *F*(3, 2386) = 15,339, *p* < 0.001, η^2^p = 0.019). A statistically significant interaction effect was also observed between sociometric type and gender (Λ = 0.991, *F*(12, 6313) = 1.801, *p* < 0.05, η^2^p = 0.003) (see Table 2).

### 3.1. Sociometric Type

The ANOVA showed significant differences in satisfaction with life *F*(3, 2393) = 5760, *p* < 0.001, η^2^p = 0.010 and cyber-aggression *F*(3, 2393) = 4.644, *p* < 0.001, η^2^p = 0.008 (see Table 3). The Bonferroni tests (α = 0.05) indicated that rejected students had lower life satisfaction scores than preferred, average and neglected students. Rejected students also reported greater involvement in cyber-aggression than preferred, controversial and average students.

### 3.2. Gender

The ANOVA showed significant differences with respect to gender in the variables psychological distress (*F*(1, 2397) = 152,748, *p* < 0.001, η^2^p = 0.060) and cyber-aggression (*F*(1,2397) = 3.878, *p* < 0.05, η^2^p = 0.002). As shown in Table 3, the girls presented higher scores than boys for psychological distress and involvement in cyber-aggression.

### 3.3. Sociometric Type, Cyber-Aggression and Gender

**Figure 1:**
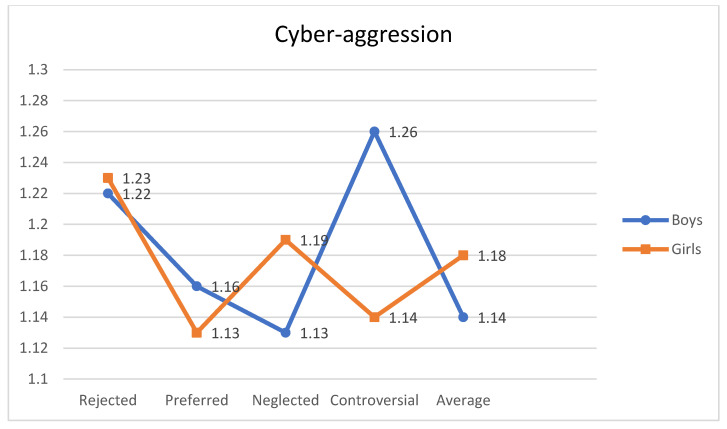
Interaction effect of sociometric types by gender and cyber-aggression.

A statistically significant interaction effect was obtained between sociometric type and gender in the cyber-aggression variable F (4, 2388) = 2.569, *p* < 0.05, η^2^p = 0.004. As shown in Table 4, the results of the post-hoc contrasts performed with the Bonferroni test (α = 0.05) indicated that the controversial boys obtained the highest scores in cyber-aggression (see Figure 1).

## 4. Discussion

The general aim of this study was to analyze the relationships between sociometric types in school (rejected, preferred, neglected, controversial and average) and psychological discomfort, life satisfaction and cyber-aggression, based on the adolescent’s gender. The results showed that satisfaction with life and cyber-aggression are significantly related to the sociometric type. A statistically significant interaction effect was also obtained for the cyber-aggression variable.

Firstly, as expected, the students with school integration problems (rejected and neglected) showed greater psychological distress and less satisfaction with life compared to the preferred, average and neglected students. However, no significant differences were found for the psychological distress variable, which was found to be equivalent in the five sociometric types. Previous research has linked rejection in school to emotional problems such as anxiety symptoms [26], depression, feelings of loneliness and stress [55], and low satisfaction with life [21,22]. It is important to highlight that this study analyzes group organization and the position occupied in same by each student based on the choices and rejections made with respect to their peers according to the criterion of group work in school [9]. In other words, rejection in school was probably attributed in this study for academic reasons and was not necessarily attributed to emotional and other aspects related to their relationships in the group (e.g., companionship or sympathy). In our opinion, this result is interesting and deserves further exploration, differentiating rejection caused by problems or difficulties in interpersonal relationships from rejection related to academic issues.

Secondly, it was observed that rejected students were more involved in cyber-aggression than preferred, controversial and average students. These results coincide with those described in previous research reporting that cyber-bullies have fewer friends and that support from friends and colleagues is an important protector against involvement in cyber-aggression [16,28]. In this sense, an interesting study performed by Wright & Li (2013) [56] reports that peer rejection could intensify negative feelings and an accumulation of anger and tension, thus increasing the risk of involvement in cyber-aggression. However, and contrary to expectations, no significant differences were observed in neglected students. This result may be due to the fact that although these adolescents are neglected in their peer group (they receive little attention from the latter and are not well known), they do not necessarily have to be socially isolated as occurs with certain rejected adolescents [9,17] and, consequently, they most likely tend to avoid potentially violent situations to avoid undermining their social network or situations of victimization.

In terms of the effect of interaction between sociometric types and gender, the results partially confirmed the third hypothesis, since only one interaction effect was obtained. The results showed that controversial boys had the highest scores in cyber-aggression, but in situations of rejection girls and boys showed high and similar involvement in this problematic behavior. Previous studies have emphasized that, although both controversial and rejected students tend to be involved in violent behavior [57,58], controversial students are generally characterized by having greater academic skills [15]. Likewise, it has been observed that girls report higher expectations of school success, academic performance, and obtain better evaluations from their peers in academic cooperation [36,59]. Therefore, it is plausible to think that girls who receive social support in school avoid becoming involved in violence to a greater extent and comply with the norms and rules of coexistence due to their desire to continue to be seen and chosen as “good group companions” [58], and due to their interest in and motivation towards school [42,60].

In terms of interaction, the findings also showed that neglected girls had higher scores than neglected boys in cyber-aggression, while neglected students did not differ, regardless of gender, so it follows that the negative impact of this painful and stressful situation is greater in neglected girls. In previous studies, it has been observed that boys are more likely than girls to engage in various forms of direct aggression, actions directly targeted at the victim (e.g., physical violence or sending harmful messages and images without the need to conceal identity) [32,60]. However, girls are more likely than boys to carry out various forms of indirect aggression, actions that allow the aggressor to hide his or her identity from the victim, sometimes even making it difficult for the victim to know that he or she has been the object of any intentional harm [61,62]. These actions include spreading rumors or telling others not to associate with the victim. It is plausible to think that such greater involvement among neglected girls may stem from anger and impotence when faced with indifference and lack of attention from their peers; this response is facilitated by the anonymity that exists in the virtual environment [63]. Future research should continue to study the role of gender and sociometric type in cyber violence, taking into account whether it is direct or indirect and whether neglected girls use online aggression as a defense mechanism or to respond to previous aggression.

Finally, it is important to highlight that the results obtained in this research should be interpreted with caution, due to the cross-sectional and correlational nature of the data. Future research incorporating the time dimension would allow us to clarify the differences obtained between the groups. Moreover, the measurement of psychological distress, satisfaction with life and cyber-aggression variables, as self-reporting measurements, may entail a certain bias and the effects of social desirability. This limitation could be resolved through the incorporation of different sources of information (peer group, teachers, family). Future research and prevention and intervention programs should take these variables into account while also considering a multidimensional perspective integrating the different types of cyber-aggressors, their integration in school and the differential role that gender socialization plays in students.

## 5. Conclusions

The findings of this research show that school integration is fundamental in the emotional well-being and psychosocial adjustment of adolescents. Thus, social acceptance enhances satisfaction with life, probably because having friends can increase self-esteem and self-concept and thus broaden one’s sphere of relationships both offline and online. In turn, having more support resources is associated with greater psychosocial well-being and adjustment. In this sense, programs should be promoted to prevent social difficulties in the school, based on the enhancement of social integration, not only in the classroom, but also online, in order to provide adolescents with the necessary resources to help them reduce the likelihood of involvement in acts of school violence, while promoting the development of a more satisfactory school experience. Finally, the use of sociometry in education professionals is recommended. This technique would provide information about each student, highlighting the ascription of each individual to a certain sociometric type and their distance from each of their classmates, and would provide highly relevant information about the group as a whole, the internal configuration of their relationships, and their degree of cohesion and coherence.

## Figures and Tables

**Table 1:** Number of cases in sociometric type groups.

Total Sample		Rejected	Preferred	Neglected	Controversial	Average	χ^2^
		*n* = 273	*n* = 188	*n* = 291	*n* = 86	*n* = 1560	
Gender							
Boys	1203	174	78	142	58	751	χ^2^(4) = 38.815 ^***^
	(50.2%)	(63.7%)	(41.5%)	(48.8%)	(67.4%)	(48.1%)	
Girls	1195	99	110	149	28	809	
	(49.8%)	(36.3%)	(58.5%)	(51.2%)	(32.6%)	(51.9%)	

Note: χ^2^: Chi-square. *** *p* < 0.001.

**Table 2:** MANOVA results for all the studied variables (5 ^a^ × 2 ^b^).

Variables		Source of Variation				
	*Λ*	*F*	*df_between_*	*df_error_*	*p*	η*^2^p*
(A) Sociometric Type ^a^	0.985	1801	12	6313	<0.001 ***	0.005
(B) Gender ^b^	0.981	15,339	3	2386	<0.001 ***	0.019
A × B	0.991	1801	12	6313	<0.05 *	0.003

Note: a: Rejected, Preferred, Neglected, Controversial, Average; b: Male, Female; *** *p* < 0.001; * *p* < 0.05.

**Table 3:** Means (Standard Deviations) of Sociometric Type, and Main Univariate *F* Values for Psychological Distress, Life Satisfaction, Cyber-aggression, and Gender.

Sociometric Types								Gender			
Variables	R	P	N	C	A	*F*(3, 793)	η^2^*p*	Girls	Boys	*F*(3, 793)	η^2^*p*
P.D.	2.46 (0.70)	2.36 (0.66)	2.38 (0.64)	2.36 (0.63)	2.39 (0.71)	0.78 n.s.	0.001	2.22 (0.65)	2.56 (0.70)	152.748 ***	0.060
L.S.	3.13 (0.78) ^b^	3.41 (0.68) ^a^	3.33 (0.75) ^a^	3.19 (0.76)	3.33 (0.75) ^a^	5.760 ***	0.010	3.33 (0.73)	3.28 (0.77)	1.952 n.s.	0.001
C	1.22 (0.32) ^a^	1.14 (0.21) ^b^	1.16 (0.22)	1.22 (0.27) ^b^	1.16 (0.26) ^b^	4.644 **	0.008	1.16 (0.25)	1.18 (0.27)	3.878 *	0.002

Note: P.D. = Psychological Distress; L.S. = Life Satisfaction; C = Cyber-aggression; R = Rejected; P = Preferred; N = Neglected; C = Controversial; A = Average; a: Rejected, Preferred, Neglected, Controversial, Average; b: Male, Female; *** *p* < 0.001; ** *p* < 0.01; * *p* < 0.05; a > b.

**Table 4:** Means (Standard Deviations), and post-hoc comparisons between sociometric types, cyber-aggression and gender.

Sociometric Type									
Variables	Gender	R	P	N	C	A	*F*(4, 2388)	η*^2^p*	Post Hoc
C	Boys	1.22 (0.32) ^1^	1.16 (0.27) ^2^	1.13 (0.19) ^3^	1.26 (0.29) ^4^	1.14 (0.23) ^5^	2.569 *	0.004	4 > 2; 9; 10 1; 4; 6 > 7 1; 4; 6; 10> 3; 5 8 > 3
	Girls	1.23 (0.33) ^6^	1.13 (0.17) ^7^	1.19 (0.24) ^8^	1.14 (0.20) ^9^	1.18 (0.28) ^10^			

Note: C = Cyber-aggression; R = Rejected; P = Preferred; N = Neglected; C = Controversial; A = Average. * *p* < 0.05.

## Data Availability

Data sharing is not applicable to this article.
